# Stress hyperglycemia ratio (SHR) and triglyceride-glucose index (TYG) associated with renal response to finerenone in diabetic kidney disease: a retrospective cohort study

**DOI:** 10.3389/fmed.2026.1802071

**Published:** 2026-06-03

**Authors:** Anqi Chen, Liting Zou, Wei Sun, Xuelian Yao, Xinfeng Qian, Fang Shao

**Affiliations:** Emergency Department, Affiliated Hospital of Jiangnan University, Wuxi, China

**Keywords:** diabetic kidney disease (DKD), finerenone, renal response, stress hyperglycemia ratio (SHR), triglyceride-glucose index (TyG)

## Abstract

**Background:**

Diabetic kidney disease (DKD) is a major complication of type 2 diabetes mellitus (T2DM) and a leading cause of end-stage kidney disease (ESKD). This study aimed to investigate the association between stress hyperglycemia ratio (SHR) and triglyceride-glucose (TyG) index with renal outcomes in patients with Chronic Kidney Disease (CKD) initiating finerenone therapy.

**Methods:**

This retrospective cohort study included 387 adults with DKD and CKD stages 1–4 who initiated finerenone therapy between January 2023 and December 2025. The primary composite renal endpoint included sustained ≥40% eGFR decline or progression to ESKD. Secondary outcomes were absolute UACR change at 12 months and annualized eGFR decline rate. Cox proportional hazards models evaluated their associations with renal outcomes, adjusted for clinical covariates.

**Results:**

The primary endpoint was reached by 62 patients (16.0%). Higher baseline SHR and TyG were associated with worse renal outcomes. In multivariable analysis, the highest SHR tertile (T3) had a 2.52-fold increased risk (95% CI 1.48–4.29; *p* = 0.001) and the highest TyG tertile (T3) had a 1.96-fold increased risk (95% CI 1.22–3.15; *p* = 0.006) compared to the lowest tertiles. Subgroup analyses showed stronger associations in advanced CKD stages. At 12 months, high-index groups had attenuated UACR reduction and accelerated eGFR decline. Adding SHR and TyG to the clinical model improved predictive performance, with a C-statistic increase from 0.70 to 0.78.

**Conclusion:**

Higher SHR and TyG values were independently associated with worse renal outcomes in patients with DKD initiating finerenone therapy. These biomarkers could enhance predictive models and inform targeted interventions.

## 1 Introduction

Diabetic kidney disease (DKD) is a major complication of type 2 diabetes mellitus (T2DM) and a leading cause of end-stage kidney disease (ESKD) worldwide (1). Despite advances in glycemic control and the use of renoprotective medications, the burden of DKD remains high, highlighting the need for more effective strategies to mitigate disease progression (2). Finerenone, a nonsteroidal mineralocorticoid receptor antagonist (MRA), has shown promise in reducing both kidney and cardiovascular risks associated with DKD. It achieves this by targeting inflammation and fibrosis mediated by the mineralocorticoid receptor (MR) (3). However, the extent of inflammation and fibrosis can vary significantly among patients, which may result in inconsistent responses to Finerenone therapy. Pivotal trials have indicated that finerenone can reduce the relative risk of composite renal outcomes, which include a sustained decline in eGFR of ≥40% or progression to ESKD, by 23%. Despite these positive findings, approximately 30% of patients demonstrate a suboptimal reduction in UACR of less than 30%, which may imply a non-response to the treatment (4). This variability in patient response highlights the importance of identifying predictive biomarkers to better stratify patient populations and personalize treatment approaches.

Insulin resistance (IR) and hyperglycemia are central to the pathogenesis of T2DM and DKD. Both acute and chronic metabolic disturbances contribute to the progression of renal disease (5). The stress hyperglycemia ratio (SHR) and the triglyceride-glucose (TyG) index are emerging biomarkers that reflect these disturbances. The SHR, defined as the ratio of admission glucose to a calculated value based on HbA1c, captures transient hyperglycemia often seen in acute stress states (6). The TyG index, calculated from fasting triglycerides and glucose levels, is a surrogate marker for IR and has been shown to predict cardiovascular and renal outcomes in various populations (7, 8). Both markers have been associated with adverse renal outcomes in patients with T2DM, but their combined impact on renal response to finerenone therapy remains underexplored.

Previous studies have demonstrated the predictive value of the SHR and TyG index for cardiovascular and renal outcomes in patients with T2DM (9, 10). However, these studies have primarily focused on single-point measurements of these biomarkers, failing to capture the dynamic changes over time. Given the variability in patient responses to finerenone, it is crucial to identify predictive biomarkers to better stratify patient populations and personalize treatment approaches. This study thus focuses on evaluating the clinical utility of SHR and TyG index in predicting renal outcomes for DKD patients undergoing finerenone therapy.

## 2 Methods

### 2.1 Study design and participants

This retrospective cohort study consecutively enrolled adults (≥18 years) with diabetic kidney disease (DKD, CKD stages 1–4) initiating finerenone therapy at our hospital between January 2023 and December 2025. Diagnoses complied with the 2020 KDIGO Clinical Practice Guidelines (11). Exclusion criteria were: (1) biopsy-proven non-diabetic kidney disease; (2) active malignancy; (3) pregnancy; (4) incomplete baseline data; (5) follow-up <12 months.

This study was approved by the Institutional Review Board of our Hospital (Approval number: KY-202602014) and conducted in accordance with the ethical standards of the Declaration of Helsinki. Individual informed consent was waived due to the retrospective and anonymized nature of the data analysis.

### 2.2 Data collection and variable definitions

Demographic, clinical, and laboratory parameters (including fasting glucose, HbA1c, triglycerides, serum creatinine, and urinary albumin-to-creatinine ratio [UACR]) were extracted from electronic medical records. The SHR was computed as admission glucose/(28.7 × HbA1c−46.7) following Roberts et al. (12). The TyG was derived as ln[fasting triglycerides (mg/dL) × fasting glucose (mg/dL)/2] (13). Estimated glomerular filtration rate (eGFR) was calculated using the CKD-EPI creatinine equation.

### 2.3 Outcome assessment

The primary composite renal endpoint comprised: (1) sustained ≥40% eGFR decline from baseline, or (2) progression to ESKD requiring renal replacement therapy. Secondary outcomes included absolute UACR change at 12 months and annualized eGFR decline rate (calculated via linear mixed models). Two independent nephrologists blinded to exposure variables adjudicated all endpoints.

### 2.4 Statistical analysis

Continuous variables were summarized as mean ± standard deviation (SD) or median (interquartile range [IQR]) based on normality testing (Shapiro–Wilk test). Categorical variables were reported as frequencies (percentages). Group comparisons used Student’s t-test, Mann–Whitney U-test, or *χ*^2^ tests as appropriate.

SHR and TyG were categorized into tertiles (T1-T3) based on their distribution in the entire cohort. The lowest tertile (T1) served as the reference group in all analyses. Cox proportional hazards models evaluated associations between SHR/TyG (as continuous per 1-SD increase and as tertiles) and the composite outcome. The primary multivariable model adjusted for age, sex, baseline eGFR, UACR, systolic blood pressure, and RAS inhibitor use.

Subgroup analyses stratified by CKD stage (G1-2: eGFR ≥60; G3a: eGFR 45–59; G3b-4: eGFR <45 mL/min/1.73m^2^) were conducted. Multiplicative interaction between SHR and TyG was tested by including their product term in Cox models. For secondary outcomes, UACR response was analyzed using ANCOVA adjusted for baseline UACR, while eGFR slopes were estimated with linear mixed-effects models.

Predictive performance was assessed via time-dependent receiver operating characteristic (ROC) analysis at 12 months, with Harrell’s C-statistic computed using bootstrap resampling (2000 replicates). All analyses were performed in R 4.3.0 (packages: survival, lme4, timeROC) with statistical significance defined as two-tailed *p* < 0.05.

## 3 Results

### 3.1 Study cohort and baseline characteristics

Between January 2023 and December 2025, 523 patients initiating finerenone therapy were screened. After applying exclusion criteria (non-diabetic kidney disease, active malignancy, pregnancy, incomplete data, or <12-month follow-up), 387 patients comprised the analytical cohort. Median follow-up was 17.8 months (IQR 13.1–21.6), with 62 patients (16.0%) reaching the composite renal endpoint (58 [93.5%] were sustained eGFR decline ≥40%, and 4 [6.5%] progressed to ESRD).

As shown in Table 1, patients experiencing the endpoint exhibited significantly higher baseline stress hyperglycemia ratio (SHR: 1.38 ± 0.31 vs. 1.04 ± 0.19; *p* < 0.001) and triglyceride-glucose index (TyG: 9.48 ± 0.69 vs. 8.87 ± 0.59; *p* < 0.001) compared to the non-event group. They also demonstrated more advanced kidney disease, including lower baseline eGFR (37.6 ± 14.2 vs. 46.3 ± 13.8 mL/min/1.73m^2^; *p* < 0.001), higher UACR (median 1,320 [IQR 605–2,640] vs. 385 [150–850] mg/g; p < 0.001), and greater prevalence of CKD stages 3–4 (85.5% vs. 64.0%; *p* = 0.001). Differences in age, sex distribution, and RAS/SGLT2 inhibitor use were non-significant.

**Table 1:** Baseline characteristics stratified by composite renal outcome.

Characteristic	Non-event (*n* = 325)	Event (*n* = 62)	*p*-value
Age (years)	63.5 ± 8.7	65.9 ± 9.1	0.061
Male, *n* (%)	187 (57.5%)	37 (59.7%)	0.77
Fasting glucose (mg/dL)	148 ± 32	172 ± 41	<0.001
HbA1c (%)	8.2 ± 1.4	9.1 ± 1.8	<0.001
Triglycerides (mg/dL)	168 (122–228)	216 (158–294)	<0.001
SHR	1.04 ± 0.19	1.38 ± 0.31	<0.001
TyG index	8.87 ± 0.59	9.48 ± 0.69	<0.001
Baseline eGFR (mL/min/1.73m^2^)	46.3 ± 13.8	37.6 ± 14.2	<0.001
UACR (mg/g)	385 (150–850)	1,320 (605–2,640)	<0.001
CKD stage 3–4, *n* (%)	208 (64.0%)	53 (85.5%)	0.001
RAS inhibitor use, *n* (%)	288 (88.6%)	55 (88.7%)	0.99
SGLT2 inhibitor use, *n* (%)	102 (31.4%)	19 (30.6%)	0.91

### 3.2 Association of SHR and TyG with composite renal outcome

In multivariable Cox regression adjusted for age, sex, baseline eGFR, UACR, systolic blood pressure, and RAS inhibitor use, both SHR and TyG independently associated with the composite renal outcome (Table 2). Patients in the highest SHR tertile (T3) had a 2.52-fold increased risk versus the lowest tertile (T1) (95% CI 1.48–4.29; *p* = 0.001). Similarly, TyG T3 conferred a 1.96-fold higher risk than TyG T1 (95% CI 1.22–3.15; *p* = 0.006). Per 1-SD increase, SHR was associated with a 67% elevated risk (HR 1.67, 95% CI 1.38–2.02; *p* < 0.001), while TyG increased risk by 51% (HR 1.51, 95% CI 1.25–1.83; *p* < 0.001).

**Table 2:** Association of SHR and TyG with composite renal outcome.

Predictor	Unadjusted HR (95% CI)	*p*-value	Adjusted HR* (95% CI)	*p*-value
SHR (per 1-SD)	1.85 (1.58–2.17)	<0.001	1.67 (1.38–2.02)	<0.001
SHR tertiles				
T1 (<0.94)	1.00 (Reference)	—	1.00 (Reference)	—
T2 (0.94–1.18)	1.61 (0.96–2.70)	0.071	1.52 (0.87–2.65)	0.14
T3 (>1.18)	3.24 (2.03–5.18)	<0.001	2.52 (1.48–4.29)	0.001
TyG (per 1-SD)	1.72 (1.45–2.04)	<0.001	1.51 (1.25–1.83)	<0.001
TyG tertiles				
T1 (<8.62)	1.00 (Reference)	—	1.00 (Reference)	—
T2 (8.62–9.25)	1.52 (0.91–2.53)	0.11	1.38 (0.82–2.33)	0.23
T3 (>9.25)	2.81 (1.76–4.49)	<0.001	1.96 (1.22–3.15)	0.006

*Adjusted for age, sex, baseline eGFR, UACR, systolic BP, and RAS inhibitor use.

### 3.3 Association of SHR and TYG with the composite endpoint in different stages of CKD

Subgroup analyses based on KDIGO CKD stages revealed a gradient effect for both indices. In CKD stages 3b–4 (eGFR <45 mL/min/1.73m^2^; *n* = 131), each 1-SD increase in SHR corresponded to a 92% higher risk (HR 1.92, 95% CI 1.52–2.43), whereas in CKD stages 1–2 (eGFR ≥60; *n* = 121), the association was non-significant (HR 1.41, 95% CI 0.97–2.06). TyG showed parallel trends (CKD 3b–4: HR 1.77, 95% CI 1.42–2.21; CKD 1–2: HR 1.38, 95% CI 0.99–1.92). Formal interaction testing using multiplicative interaction terms (CKD stage × SHR and CKD stage × TyG) in the Cox models showed a non-significant interaction for either SHR (P-interaction = 0.18) or TyG (P-interaction = 0.24), suggesting that the associations of these biomarkers with renal outcomes were consistent across CKD stages (Table 3).

**Table 3:** Association of SHR and TYG with the composite endpoint in different stages of CKD.

CKD stage	eGFR range (mL/min/1.73m^2^)	*n*	SHR HR (95% CI)	*p*-value	TyG HR (95% CI)	*p*-value
Stage 1–2	≥60	121	1.41 (0.97–2.06)	0.072	1.38 (0.99–1.92)	0.058
Stage 3a	45–59	135	1.65 (1.27–2.14)	<0.001	1.49 (1.18–1.88)	0.001
Stage 3b–4	<45	131	1.92 (1.52–2.43)	<0.001	1.77 (1.42–2.21)	<0.001

All models adjusted for age, sex, baseline UACR, systolic BP, RAS inhibitor use.

### 3.4 Interaction between SHR and TyG

A significant multiplicative interaction existed between SHR and TyG (interaction term HR 1.28, 95% CI 1.09–1.50; *p* = 0.004). Patients with both SHR and TyG above median values (*n* = 97) had a 3.21-fold increased risk (95% CI 1.89–5.46) versus those below both medians (*n* = 101), indicating synergistic effects (Table 4).

**Table 4:** Interaction between SHR and TyG.

Exposure group	*n*	Events (%)	Adjusted HR* (95% CI)	*p*-value
Low SHR + Low TyG^†^	101	11 (10.9%)	1.00 (Reference)	—
Low SHR + High TyG	89	16 (18.0%)	1.72 (0.98–3.02)	0.059
High SHR + Low TyG	92	19 (20.7%)	2.04 (1.18–3.53)	0.011
High SHR + High TyG	97	33 (34.0%)	3.21 (1.89–5.46)	<0.001
Interaction term			1.28 (1.09–1.50)	0.004

*Adjusted for age, sex, baseline eGFR, UACR, systolic BP, RAS inhibitor use.

^†^Low/High defined by median values: SHR median = 1.12, TyG median = 9.05.

P-interaction for CKD stage × SHR = 0.18; P-interaction for CKD stage × TyG = 0.24.

### 3.5 Secondary outcomes and predictive performance

At 12 months, UACR reduction was attenuated in high-index groups (Table 5): SHR T3 showed a − 19.8% change (95% CI − 25.6 to −13.3) versus −36.2% (−41.5 to −30.1) in T1 (*p* < 0.001). Similarly, TyG T3 had −22.4% (−28.2 to −16.0) versus −33.7% (−38.9 to −28.0) in T1 (*p* = 0.003). eGFR decline accelerated in SHR T3 (−4.05 mL/min/year, 95% CI − 5.21 to −2.89) and TyG T3 (−3.87 mL/min/year, −4.98 to −2.76).

**Table 5:** Secondary outcomes: treatment response at 12 months.

Index/group	ΔUACR % (95% CI)	*p*	eGFR slope (mL/min/yr) (95% CI)	*p*
SHR tertiles				
T1 (*n* = 129)	−36.2 (−41.5 to −30.1)	Reference	−2.31 (−3.15 to −1.47)	Reference
T2 (*n* = 128)	−28.7 (−34.0 to −22.9)	0.021	−3.14 (−4.08 to −2.20)	0.042
T3 (*n* = 130)	−19.8 (−25.6 to −13.3)	<0.001	−4.05 (−5.21 to −2.89)	0.001
TyG tertiles				
T1 (*n* = 128)	−33.7 (−38.9 to −28.0)	Reference	−2.45 (−3.30 to −1.60)	Reference
T2 (*n* = 127)	−27.9 (−33.3 to −22.0)	0.033	−3.27 (−4.22 to −2.32)	0.038
T3 (*n* = 132)	−22.4 (−28.2 to −16.0)	0.003	−3.87 (−4.98 to −2.76)	0.006

ΔUACR, percentage change from baseline; eGFR slope, annual decline rate.

Time-dependent ROC analysis demonstrated improved prediction with SHR/TyG: the clinical model (age/sex/eGFR/UACR/SBP/RASi) had a 12-month C-statistic of 0.70 (95% CI 0.63–0.77), which increased to 0.75 (0.69–0.81) after adding SHR and reached 0.78 (0.72–0.84) with both indices (Table 6).

**Table 6:** Predictive performance of SHR and TyG.

Model	Harrell’s C-statistic (95% CI)	ΔC vs. clinical model*	*P* for comparison
Clinical model*	0.70 (0.63–0.77)	Reference	—
Clinical model + SHR	0.75 (0.69–0.81)	+0.05	0.002
Clinical model + TyG	0.73 (0.66–0.80)	+0.03	0.019
Clinical model + SHR + TyG	0.78 (0.72–0.84)	+0.08	<0.001

*Clinical model: age, sex, baseline eGFR, UACR, systolic BP, RAS inhibitor use.

*C-statistic calculated at 12-month follow-up.

## 4 Discussion

This study investigated the association between the SHR and the TyG index with renal outcomes in patients with DKD initiating finerenone therapy. Our findings demonstrate that higher SHR and TyG values are independently associated with worse renal outcomes, including sustained eGFR decline and progression to ESKD. Higher SHR suggests acute metabolic stress impacts renal prognosis, while elevated TyG indicates chronic insulin resistance and lipid abnormalities driving renal damage. Both biomarkers provide unique information on metabolic disturbances in DKD pathophysiology and help gauge disease severity and progression risk.

The renoprotective effects of finerenone are primarily mediated through its ability to antagonize the MR, thereby inhibiting inflammation and fibrosis pathways. However, the extent of these pathological processes varies widely among patients. In patients with more severe inflammation or fibrosis, finerenone may exert a more pronounced therapeutic effect, as evidenced by the significant association between baseline SHR/TyG and renal outcomes in our study (14, 15). Conversely, in patients with less severe inflammation or fibrosis, the therapeutic effect of finerenone might be less apparent. This variability in therapeutic response highlights the need to better understand the interplay between finerenone and the underlying pathological processes in individual patients. The therapeutic response to finerenone may also be influenced by individual patient differences, including comorbidities, and drug interactions. Patients with comorbidities such as cardiovascular diseases or metabolic diseases, or those using other medications like ACEIs, ARBs, or SGLT2 inhibitors, may show different responses to finerenone. These factors collectively emphasize the importance of adopting a personalized approach to finerenone therapy (16).

The SHR and TyG index are easily calculable from routine clinical data, making them practical biomarkers for risk stratification in clinical practice. The SHR captures acute metabolic disturbances, reflecting transient hyperglycemia that may occur in response to stress or illness (17). This transient hyperglycemia can exacerbate renal injury, particularly in patients with pre-existing CKD (18). The mechanisms underlying the association between SHR and renal outcomes are multifaceted. Acute hyperglycemia, as indicated by SHR, can lead to the activation of the renin-angiotensin-aldosterone system (RAAS), which exacerbates renal injury through increased glomerular pressure and proteinuria (19). The TyG index, on the other hand, reflects chronic insulin resistance, a key driver of both T2DM and CKD progression (20). Chronic insulin resistance, as reflected by the TyG index, can lead to increased oxidative stress and inflammation, which contribute to renal fibrosis and glomerulosclerosis (21). Additionally, insulin resistance is associated with dyslipidemia, which can lead to the accumulation of lipids in renal tissues, further contributing to renal injury (22). By incorporating these biomarkers into clinical models, clinicians can better identify patients who may benefit from more aggressive interventions, such as early initiation of finerenone therapy.

Our study also found a significant multiplicative interaction between SHR and TyG, indicating that patients with both high SHR and high TyG values have a synergistically increased risk of renal outcomes. This finding suggests that the combined effect of acute and chronic metabolic disturbances may be more detrimental than either alone (23). This interaction highlights the importance of addressing both acute and chronic metabolic derangements in the management of patients with DKD. Our study also found a significant multiplicative interaction between SHR and TyG. This finding is consistent with previous studies demonstrating the predictive value of these biomarkers for cardiovascular and renal outcomes in patients with T2DM (24–26). For instance, a study by Shang et al. suggested that the TyG index was associated with the development of diabetic nephropathy, characterized by increased urinary albumin excretion and declining eGFR (27). Similarly, research by Liu et al. demonstrated that the TyG index predicts the progression of CKD in patients with type 2 diabetes (28). Our study extends these findings by showing that both SHR and TyG index are independently associated with renal outcomes in patients with DKD initiating finerenone therapy. The addition of SHR and TyG to the clinical model significantly improved its predictive performance. This improvement underscores the value of these biomarkers in enhancing risk prediction models.

Several limitations should be acknowledged. First, this was a retrospective cohort study, which limits our ability to establish causality. Future prospective studies or randomized controlled trials are needed to confirm our findings. Second, our study population was limited to patients initiating finerenone therapy, which may not be representative of all patients with DKD. Third, we did not account for changes in SHR and TyG over time, which could provide additional insights into renal outcomes. Lastly, our study was conducted at a single center, and external validation in other populations is needed to generalize our findings.

## 5 Conclusion

In conclusion, our study demonstrates that higher SHR and TyG values are independently associated with worse renal outcomes in patients with DKD initiating finerenone therapy. These biomarkers enhance the predictive performance of existing clinical models and may help identify patients at higher risk for renal disease progression.

## Data Availability

The original contributions presented in the study are included in the article/supplementary material, further inquiries can be directed to the corresponding author.
